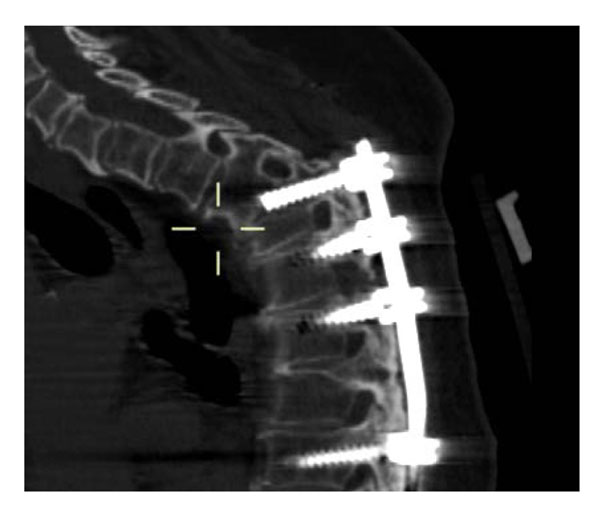# Is there a correlation between pelvic incidence and proximal junctional kyphosis (PJK) after surgery for adult scoliosis?

**DOI:** 10.1186/1748-7161-10-S1-O53

**Published:** 2015-01-19

**Authors:** Stefano Giacomini, Mario Di Silvestre, Francesco Lolli, Francesco Vommaro, Konstantinos Martikos, Elena Maredi, Andrea Baioni, Tiziana Greggi

**Affiliations:** 1 Deformities of Spine Surgery, Rizzoli Orthopaedic Institute, Bologna, Italy

## Background

The proximal junctional kyphosis (PJK) occurs from 17% to 39% after posterior arthrodesis for spinal deformity. The aim of this study is to verify a possible correlation between PJK and pelvic incidence (PI) in the treatment of adult scoliosis.

## Materials and methods

78 consecutive patients (63 women - 15 men) were included in the study, mean age of 66 years (range 60-77), surgically treated at our Division between 2000 and 2005. In all cases the diagnosis was idiopathic scoliosis, with positive sagittal imbalance. In 29 cases (37.2 %) a previous arthrodesis was performed. All patients were treated with posterior arthrodesis with pedicle instrumentation and a pedicle subtraction osteotomy (PSO) in 17 cases and Smith Petersen osteotomy ( SPO ) at multiple levels in 25 cases. The clinical and radiographic questionnaires (Oswestry, VAS) filled in before and after surgery and at final follow-up were evaluated.

## Results

After a mean follow-up of 3.8 years (range, 2-8) 18 cases of PJK occurred (23.7 %). In case of "short" synthesis (upper instrumented vertebra " UIV " between T10 and L1), the incidence increased to 50% (9 cases). PJK always occurred within 2 months after primary surgery, all cases symptomatic and evolutionary and required a surgery recovery. Considering the value of pelvic incidence (PI), patients were divided into 2 groups: Group A (IP<55 ° : 48 cases) and Group B (PI > 55 ° : 30 cases). In Group B we found a greater loss of lumbar lordosis (19.2 ° vs. 7.7 °), sagittal balance correction (28.7 % vs. 8.4 %) and we found also a higher incidence of PJK (40% vs. 12.5 %) at final follow-up.

## Conclusions

The PJK is an important and not so rare complication in spinal deformities surgery. Our data showed that a "short" synthesis (UIV between T10 and L1) and a high PI (> 55 °) is associated with a high risk of developing PJK. Patients with high pelvic incidence, thus requiring a more aggressive surgical treatment, such as with osteotomies, to achieve greater lumbar lordosis and correct sagittal balance.

**Figure 1:**
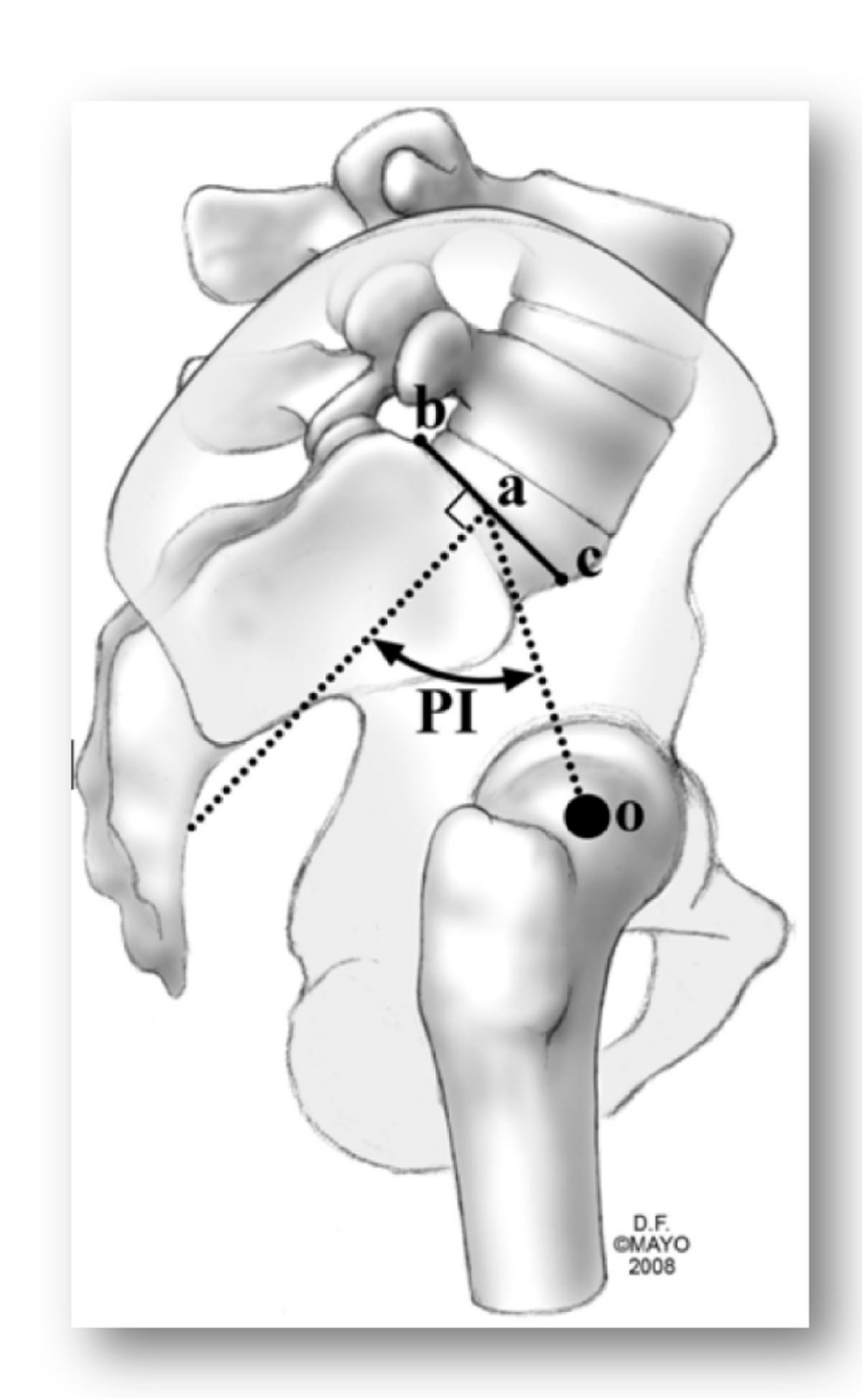


**Figure 2:**